# circ_0061265 competitively binds to microRNA-885-3p to promote the development of gastric cancer by upregulating AURKA expression

**DOI:** 10.1186/s12935-022-02646-3

**Published:** 2022-09-05

**Authors:** Qian Fei, Yuhe Lin, Mi Zhang, Jinshuai Guo, Yuan Liang

**Affiliations:** 1 Department of Oncology, Shengjing Hospital of China Medical University, Shenyang, 11021 People’s Republic of China; 2 Department of General Surgery, The Fourth Affiliated Hospital of China Medical University, Shenyang, 110032 People’s Republic of China; 3 Medical Oncology Department of Thoracic Cancer (2), Cancer Hospital of China Medical University, Liaoning Cancer Hospital & Institute, No. 44, Xiaoheyan Road, Dadong District, Shenyang, 110042 Liaoning Province People’s Republic of China

**Keywords:** circ_0061265, miRNA-885-3p, AURKA, ceRNA network, Gastric cancer, Bioinformatics analysis

## Abstract

**Background:**

Circular RNAs (circRNAs) represent a class of newly identified transcripts that act as competing endogenous RNAs (ceRNAs) to modulate gene expression by competing for the shared microRNAs (miRNAs) in humans. In this study, we set out to investigate the role of the circRNA-miRNA-mRNA ceRNA network in gastric cancer (GC).

**Methods:**

A differential analysis on GC-related circRNAs, miRNAs and mRNAs was performed utilizing the R language “limma” package, followed by GO and KEGG enrichment analyses. The Cytoscape visualization software was used to construct the circRNA-miRNA-mRNA ceRNA network. RT-qPCR, Western blot assay, immunohistochemistry, RNA pull down, RIP and dual luciferase gene reporter assay were conducted to verify the expression of the related circRNA, miRNA and mRNA and their interaction in GC tissues and cells.

**Results:**

The bioinformatics analysis screened 13 circRNAs, 241 miRNAs and 7483 mRNAs related to GC. Ten DEmRNAs (AURKA, BUB1, CCNF, FEN1, FGF2, ITPKB, CDKN1A, TRIP13, KNTC1 and KIT) were identified from the constructed PPI network and module analysis, among which AURKA was the most critical. A circ_0061265-miRNA-885-3p-AURKA ceRNA network was constructed. In vitro cell experiment demonstrated significantly upregulated circ_0061265 and AURKA, but downregulated miR-885-3p in GC. Moreover, circ_0061265 promoted the occurrence of GC by competitively binding to miRNA-885-3p to regulate AURKA expression.

**Conclusion:**

Our work validated that circ_0061265 may increase AURKA expression by competitively binding to miRNA-885-3p, thereby promoting GC development.

**Supplementary Information:**

The online version contains supplementary material available at 10.1186/s12935-022-02646-3.

## Background

Gastric cancer (GC) is amongst the most prevalent malignancies, with more than 1 million newly diagnosed cases globally and annually [1]. Despite the marked change in diagnosis and prevention, GC still ranked sixth in incidence and second in mortality on a global scale in 2018 [2]. GC is a complex, heterogeneous disease, with established risk factors from diet and lifestyle, such as *Helicobacter pylori* infection, diet, smoking, and obesity [3] to genetic mutation and instability [4], such as E-cadherin gene, PALB2, BRCA1, and RAD51C mutations [5, 6]. Laparoscopy-assisted distal gastrectomy and conventional open distal gastrectomy have been suggested as standard treatment options for GC at an early stage [7]. GC at the early stage is generally asymptomatic, whilst many patients are observed to be at the advanced stage at the time of diagnosis, on which the tumor is inoperable [8]. Thus, finding possible biomarkers for treating GC is of great importance.

Circular RNAs (circRNAs), referred to as a class of highly stable and conserved group of transcripts, can shape a closed continuous loop and are produced from non-sequential back-splicing of precursor mRNAs (pre-mRNAs) in highly diverged eukaryotes [9]. Plentiful circRNAs have been suggested to modulate diverse biological processes, including cancer differentiation, development and progression, as microRNA (miRNA) sponges and positive regulators of parental gene transcription [10]. It has been previously reported that circRNAs play an important role in GC [11]. In the present study, our bioinformatics analysis discovered circ_0061265 as a crucial differentially expressed (DE) circRNA in GC. A competing endogenous RNA (ceRNA) network indicates that transcripts, including circRNAs, their binding sites on miRNAs and compete for the control in a post-transcriptional fashion, which is becoming a new paradigm of lncRNA regulation [12]. Of note, circRNA-miRNA-mRNA ceRNA networks have been highlighted to exert important functions on GC development [13, 14]. Strikingly, microRNA-885-3p (miRNA-885-3p) was revealed to suppress the GC cell proliferation and metastasis by downregulating cyclin-dependent kinase 4 expression at the post-transcriptional level [15]. Interestingly, it was found that miRNA-885-3p could result in downregulation of Aurora kinase A (AURKA), thereby inhibiting docetaxel chemoresistance in lung adenocarcinoma [16]. AURKA is regarded as a proto-oncogenic mitotic kinase often upregulated in human epithelial tumors such as breast and ovarian cancers [17]. Intriguingly, overexpressed AURKA was found in patients with GC, which was accountable for metastasis of this malignancy [18]. Besides, suppression of AURKA could block the STAT3 pathway to diminish GC cell survival [19]. Considering the above reports, we conducted the current study aiming at exploring whether the circ_0061265-miRNA-885-3-AURKA ceRNA network affects the development of GC.

## Materials and methods

### Ethical approval

The study was carried out under the approval of Ethics Committee of Cancer Hospital of China Medical University, Liaoning Cancer Hospital & Institute.

### Acquisition of expression profiles

The GC-related circRNA microarray was obtained from the GEO database. Screening was performed from the construction of the library to August 2019, and the GSE78092 microarray was yielded, which contains 3 GC tissues and 3 adjacent normal tissues. RNA-seq data of GC were downloaded from The Cancer Genome Atlas (TCGA) database. miRNAseq and mRNAseq data were downloaded using a data transfer tool provided by GDC Apps. The miRNA sequencing data included 452 GC tissues and 45 adjacent normal tissues, and the mRNA sequencing data included 381 GC tissues and 32 adjacent normal tissues.

### Differential analysis

Limma package was used to screen differentially expressed (DE) circRNAs (DEcircRNAs), with |log_2_ (fold change)|> 2.5 and adjusted *p* value < 0.01 serving as the screening criteria. In addition, edgeR software package was utilized to screen DEmiRNAs and DEmRNAs, with the threshold set at |log_2_ (fold change) > 1 and adjusted *p* value < 0.05.

### Construction of ceRNA network

miRNA binding sites were predicted through the circRNA Interactome database (https://circinteractome.nia.nih.gov/). Based on the data from the TCGA database, the significantly upregulated target miRNAs of circRNAs were intersected with the significantly downregulated DEmiRNAs, and the significantly downregulated target miRNAs of circRNAs were intersected with the significantly upregulated DEmiRNAs. The overlapping ones obtained through the intersection were used as candidate mRNAs. Furthermore, the significantly upregulated candidate mRNAs were compared with the significantly downregulated DEmRNAs in TCGA, and the significantly downregulated candidate mRNAs with the significantly upregulated DEmRNAs in TCGA. The circRNA-miRNA pairs and miRNA-mRNA pairs were combined to construct a circRNA-miRNA-mRNA regulatory network. Finally, the network was developed and visualized via the Cycloscape v3.6.1 software.

### Protein–protein interaction (PPI) network construction and topological analysis

The interaction between DEmRNAs was evaluated using the String database (https://string-db.org/) and a PPI network was constructed. A combined score > 0.4 was used as the threshold criterion for the PPI network and a node degree of > 6 for screening hub genes. The MCC network topology algorithm in the cytoHubba application program was utilized to predict the top 10 hub genes from the PPI network, and the Cytoscape software was used to visualize the interactive network.

### Gene ontology (GO) and Kyoto encyclopedia of genes and genomes (KEGG) functional enrichment analyses

With the aim of evaluating the function of DEmRNAs in the ceRNA network on the occurrence and development of GC, the clusterProfiler package of R software was used for GO and KEGG functional analyses on the 10 identified DEmRNAs. *p* < 0.05 was set as the threshold standard.

### Clinical sample collection

GC and adjacent normal gastric tissues (≥ 5 cm from the tumor edge) were acquired from 28 patients with GC who underwent surgical treatment in Cancer Hospital of China Medical University, Liaoning Cancer Hospital & Institute from December 2015 to December 2016. All patients were followed-up for 3 years. GC patients with complete clinical data and no other complications who didn’t receive radiotherapy, chemotherapy, or immunotherapy before surgery were included. Patients were excluded from this research if they had other tumors. All tissues were stored in a refrigerator at -80℃ for later use.

### Hematoxylin and Eosin (H&E) staining

The tissue specimens were washed with physiological saline, fixed in 4% paraformaldehyde for 30–50 min, washed with water, dehydrated, cleared, waxed, embedded, and sectioned. The tissue section was placed on a glass slide, dried in a thermostat at 45 °C, deparaffinized, treated with alcohol (high-concentration to low-concentration) and then washed with distilled water for 5 min. The section was stained with hematoxylin (PT001, purchased from Shanghai Bogoo Biotechnology Co., Ltd., Shanghai, China) for 5 min, rinsed in running water for 3 s, differentiated with 1% hydrochloric acid ethanol for 3 s, stained with 5% eosin solution for 3 min, dehydrated, cleared, and mounted, followed by observation under a microscope.

### Cell treatment

The human GC cell line (NCI-N87) purchased from Shanghai Zhong Qiao Xin Zhou Biotechnology Co., Ltd. (ZQ0060, Shanghai, China) was incubated in a 5% CO_2_ incubator (saturated humidity. 37 °C) with 10% fetal bovine serum (12483020, Gibco-Invitrogen, Waltham, MA, USA)-containing Rosewell Park Memorial Institute-1640 (RPMI-1640, 11875127, Gibco-Invitrogen, Carlsbad, CA, USA) supplemented with 100 U/ml penicillin and 100 mg/ml streptomycin (15140 ~ 122, GIBCOBRL, Life Technologies, Gaithersburg, MD, USA/InviGROND).

GC cells in the logarithmic growth phase underwent transfection with negative control (NC) mimic, miR-885-3p mimic, short hairpin RNA (si)-NC + inhibitor NC (co-transfected with NC of circ_0061265 silencing and NC of miR-885-3p inhibitor), si-circ_0061265 + inhibitor NC (co-transfected with specific circ_0061265 silencing vector and NC of miR-885-3p inhibitor), si-NC + miR-885-3p inhibitor (co-transfected with NC of circ_0061265 silencing and miR-885-3p inhibitor plasmid), si-circ_0061265 + miR-885-3p inhibitor (co-transfected with specific circ_0061265 silencing vector and miR-885-3p inhibitor plasmid), si-NC, si-circ_0061265, si-AURKA, oe-NC, or oe-circ_0061265. The above vectors/plasmids were constructed on the basis of pSilencer 4.1-CMV neo (G418 resistance) vector (VT1395, Unibio) or Pegfp-N1 (G418 resistance) (VT1110, Unibio) and by Shanghai Sangon Co., Ltd. (Shanghai, China). Briefly, 1 × 10^6^ cells were treated with 50 μM miR-885-3p mimic/inhibitor, si-circ_0061265 or NC in 1 μl of Lipofectamine™ 2000 reagents (11668019, Invitrogen, Carlsbad, CA, USA) according to the instructions.

### Reverse transcription-quantitative polymerase chain reaction (RT-qPCR)

Total RNA was extracted from tissues or cells using Trizol (10296010, Invitrogen) as per the manufacturer's protocol. The concentration, purity and integrity of the obtained RNA were determined through Nano-Drop ND-1000 spectrophotometry and 1% agarose gel electrophoresis. The primers were synthesized by Sangon (Shanghai, China) (Additional file 1: Table S1). Using TaqMan MicroRNA reverse transcription kits (4366596, Applied Biosystems, Carlsbad, CA, USA) and specific RT primers of miRNA First Strand cDNA Synthesis (Tailing Reaction) (GS0150, Biolab, China), miRNA specific complementary DNA (cDNA) was synthesized. The expression of miR-885-3p was determined with TaqMan miRNA Assay kits. The expression of miR-885-3p was standardized by U6. Based on the instructions of the reverse transcription kits (TransGen Biotech, Beijing, China), the cDNA template was synthesized via reverse transcription reaction in a PCR amplification instrument. Real time RT-qPCR was performed using a fluorescent qPCR (ABI company, Oyster Bay, NY, USA). With GAPDH serving as the internal reference, and the relative expression of target gene was calculated by relative quantitative method (2^−∆∆Ct^).

### Dual luciferase gene reporter assay

The bioinformatics website predicted that circ_0061265 could bind with miR-885-3p. Artificially synthesized circ_0061265 or AURKA 3’UTR gene fragments were introduced into pMIR-reporter (Promega, Madison, WI, USA). Subsequently, the complementary sequence mutation site of the seed sequence was designed on the basis of wild type (WT) AURKA or circ_0061265 and then constructed into the pMIR-reporter plasmid. The correctly sequenced WT and mutant type (MUT) luciferase reporter plasmids were co-transfected with NC mimic and miR-885-3p mimic into NCI-N87 cells (Bnbiotech, Shanghai, China) for 48 h. Thereafter, the luciferase activity was detected by means of Dual-Luciferase Reporter Assay System (Promega).

### Western blot analysis

Total protein was subjected to extraction from GC tissues with the help of RIPA lysate (P0013B, Beyotime), with protein concentration subsequently determined using BCA kits (20201ES76, Yeasen BioTechnologies co., Ltd., Shanghai, China). After being separated by means of PAGE, the protein (50 µg) was transferred to a polyvinylidene fluoride membrane (IPVH85R, Millipore, Darmstadt, Germany). Thereafter, the membrane was blocked with 5% BSA at the ambient temperature for duration of 1 h and probed with primary antibodies against AURKA (Abcam, Cambridge, UK, ab1287, rabbit anti-human, 1: 4000), and GAPDH (Abcam, ab8245, mouse anti-human, 1: 5000), followed by overnight incubation at 4℃. Following development with enhanced chemiluminescence reagents, the protein was quantified with ImageJ 1.48u software, the analytical results of which was indicated by gray value ratio of target protein to internal reference protein was calculated.

### RNA-pull down

NCI-N87 was transfected with 50 nM biotin-labeled WT-bio-circ_0061265 and MUT-bio-circ_0061265. After 48 h, the cells were collected, washed with PBS and incubated for 10 min in a specific lysis buffer (Ambion, Austin, Texas, USA). The lysate was incubated with M-280 streptavidin magnetic beads (S3762, Sigma, USA) pre-coated with RNase-free BSA and yeast tRNA (TRNABAK-RO, Sigma, USA). The beads were incubated at 4 °C for 3 h, washed twice with pre-chilled lysis buffer, three times with low-salt buffer, and once with high-salt buffer. The bound RNA was purified by Trizol, and then the miR-885-3p enrichment was detected by RT-qPCR.

### RIP assay

The binding of circ_0061265 and miR-885-3p to AGO2 protein was assayed according to the Magna RIP RNA-Binding Pretein Immunoprecipitation kit (Millipore, USA). After pre-cooled PBS washing, the cells were lyzed using an equal volume of RIPA lysis buffer (P0013B, Beyotime) in an ice bath for 5 min, and centrifuged at 14,000 rpm for 10 min at 4 °C followed by collection of the supernatant. Part of the cell extract was taken as input, and part was incubated with antibody for co-precipitation. Specifically, 50 µL magnetic beads were washed and resuspend in 100 µL RIP Wash Buffer, and 5 µg antibody was added for binding according to the grouping. Then, the magnetic bead-antibody complex was washed, resuspended in 900 µL RIP Wash Buffer, and incubated with 100 µL cell extract at 4 °C overnight. The sample was placed on the magnetic stand to collect the magnetic bead-protein complex. The sample and Input were digested with proteinase K and RNA was extracted for subsequent PCR detection. The antibody used in RIP was: AGO2 (ab32381, 1:50, Abcam, UK) and IgG (1:100, ab109489, Abcam, UK, NC).

### Cell counting kit-8 (CCK-8) assay

CCK-8 kit (C0037, Beyotime, Shanghai) was adopted for cell proliferation detection. Briefly, cells were seeded into the 96-well-plate (1500 cells/well) with 3 parallel well prepared for each group. Optical density (OD) value at 450 nm was detected every 24 h.

For cell viability assessment, cells were seeded into the 96-well-plate (1500 cells/well). After 24 h, cells were cultured with culture medium containing cisplatin of varying concentrations for 48 h, followed by cell viability detection.

### Flow cytometry

Cells were seeded in 6-well plates at 2 × 10^5^ cells/well. After 24 h of treatment, cells were washed once with 4 °C pre-cooled PBS, digested with trypsin, and collected in 15 mL centrifuge tubes for centrifugation at 800 g with the supernatant discarded. According to the instructions of the cell apoptosis detection kit (556547, BD Bioscience, USA), cells were resuspended in 500 μL binding buffer, and incubated with 5 μL AnnexinV-FITC and 5 μL PI in the dark for 15 min. Cell apoptosis was detected by flow cytometry (BD FACSCalibur).

### Transwell assay

GC cells were digested, washed twice with PBS, and resuspended in serum-free medium DMEM (Gibco, USA). A total of 5 × 10^4^ cells were seeded into the upper chamber of a 24-well chamber with 8.0 μm pores. DMEM medium containing 20% FBS was added to the lower layer of the chamber and incubated in a 37 °C, 5% CO_2_ incubator. After 48 h, the cells were fixed with methanol for 30 min and then stained in 0.05% crystal violet (G1062, Solarbio, China) for 5 min. Migrated cells were counted and photographed.

### Statistical analysis

Statistical analysis for all the data in the current study was implemented using SPSS 21.0 software (IBM Corp, Armonk, NY, USA). Measurement data were derived from three independently repeated experiments and displayed as a form of mean ± standard deviation. Comparisons between GC and adjacent normal tissues were implemented by paired t-test, while other two-group comparisons were conducted by unpaired *t*-test. One-way analysis of variance (ANOVA) combined with Tukey's post hoc test was utilized for comparing data among multiple groups. Data among multiple groups at varied time points were compared by means of repeated measures ANOVA, followed by Bonferroni tests. Pearson correlation analysis was used for observing the correlation of indicators. *p* < 0.05 referred to statistically significant difference.

## Results

### DEcircRNAs, DEmiRNAs, and DEmRNAs in GC samples were obtained through bioinformatics analysis

**Fig. 1:**
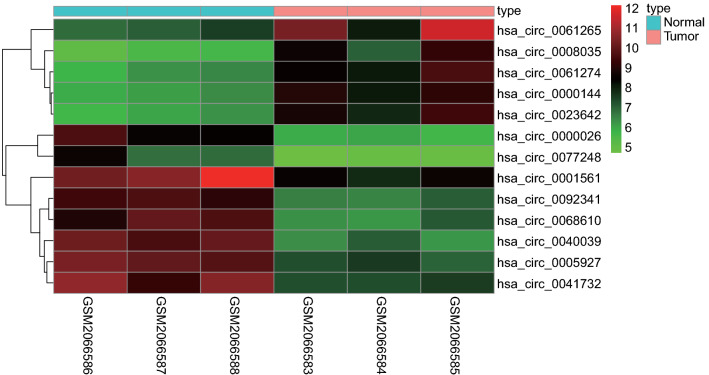
The heatmap for the 13 DEcircRNAs in GC and adjacent normal gastric tissues obtained from the GSE78092 dataset

**Fig. 2:**
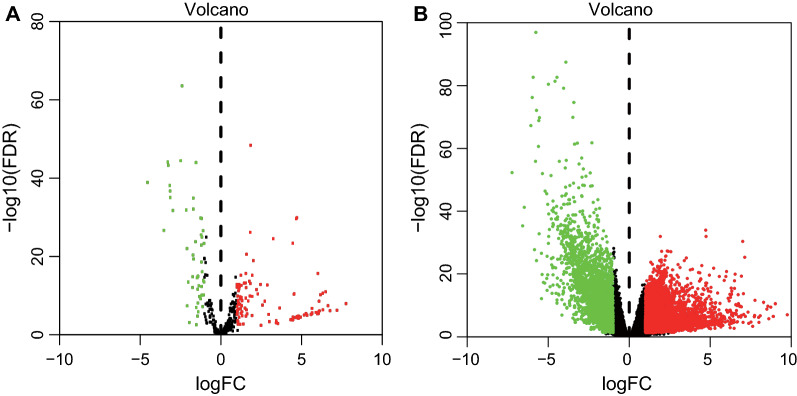
Volcano maps for DEmiRNAs (**A**) and DEmRNAs (**B**) in GC and 45 normal gastric tissue samples

The circRNA microarray data of GC and normal tissues were retrieved from the GEO database, and GSE78092 dataset was obtained. Next, differential analysis was performed using Limma package, which identified 13 DEcircRNAs (5 upregulated circRNAs and 8 downregulated ones) (Fig. 1). The information of the 13 DEcircRNAs is detailed in Additional file 1: Table S2 and their structures are shown in Additional file 2: Fig. S1. Furthermore, the sequencing data of miRNAs and mRNAs (counts) in GC and adjacent normal tissues were obtained from the TCGA database. Subsequent differential analysis using edgeR package screened 241 DEmiRNAs (180 upregulated miRNAs and 61 downregulated ones) and 7483 DEmRNAs (4474 upregulated miRNAs and 3009 downregulated ones) (Fig. 2A, B).

### Construction of circRNA-miRNA-mRNA ceRNA networks of GC based on targeting relationship prediction

**Fig. 3:**
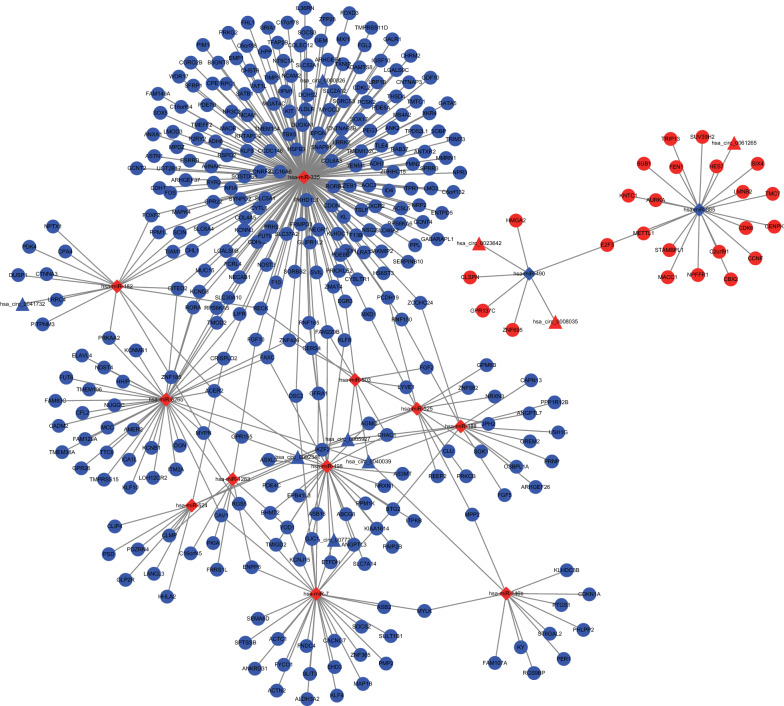
A circRNA-miRNA-mRNA ceRNA network of GC is constructed, consisting of 9 DEcircRNAs, 13 DEmiRNAs, and 339 DEmRNAs. Triangles indicate circRNAs, diamonds indicate miRNAs, and ellipses indicate mRNAs. Nodes highlighted in red indicate upregulation and those highlighted in blue indicate downregulation

In order to better understand the role of circRNAs and miRNAs in the ceRNA network of GC, a circRNA-miRNA-mRNA (ceRNA) network was established. Thirteen circRNAs and 146 circRNA-miRNA pairs were retrieved from the Circinteractome database. After intersection with DEmiRNAs, 16 circRNA-miRNA pairs of were obtained, including 9 circRNAs and 13 DEmiRNAs. In addition, a total of 2920 intersecting mRNAs were predicted in the miRTarBase and TargetScan databases. The 2920 mRNAs were overlapped with 7483 DEmRNAs, which screened 339 common DEmRNAs to construct the ceRNA network. Based on these circRNA-miRNA pairs and miRNA-mRNA pairs, a ceRNA network containing 9 ceRNA nodes, 13 miRNA nodes and 339 mRNA nodes in GC was constructed (Fig. 3).

### Reconstruction of circRNA-miRNA-mRNA in sub-network

**Fig. 4:**
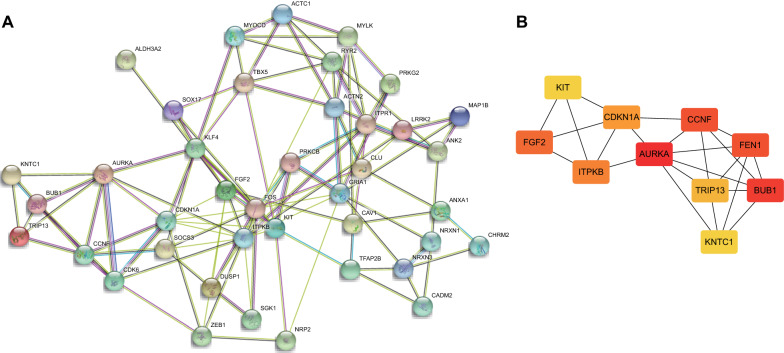
The PPI network is constructed based on DEmRNAs in GC tissue samples. **A** The PPI network of 339 genes composed of 40 nodes and 111 edges. **B** The PPI network of 10 DEmRNAs extracted from (**A**). As the log_2_ (fold change) changes, the node color gradually changes from yellow to red

**Fig. 5:**
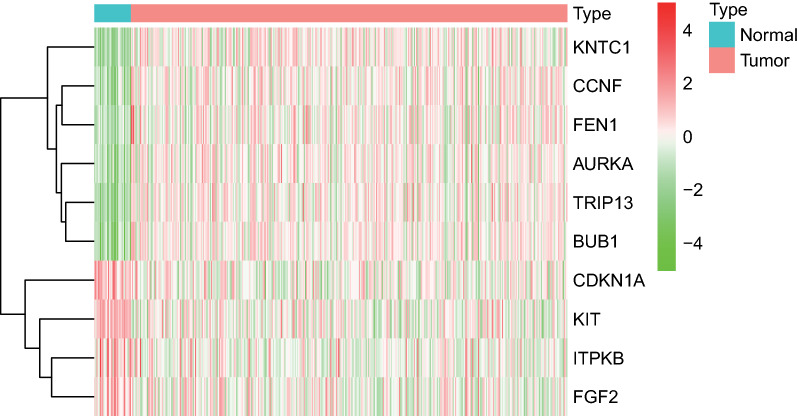
The heatmap for the 10 DEmRNAs in the mRNA microarray dataset of GC

**Fig. 6:**
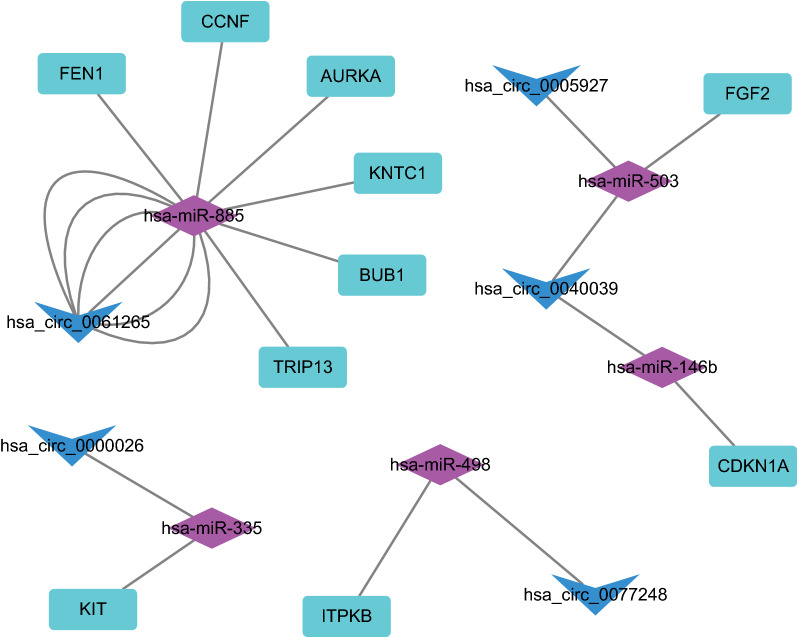
A circRNA-miRNA-mRNA ceRNA network of GC is constructed, consisting of 5 DEcircRNAs, 5 DEmiRNAs, and 10 DEmRNAs. The Vs, diamonds and rounded rectangles indicate circRNAs, miRNAs and mRNAs, respectively

A DEmRNA-based PPT network was established, involving 40 nodes and 111 edges (PPI enrichment *p* value < 1.0e^−16^) (Fig. 4A). Subsequently, MCC network topology algorithm in the cytoHubba application program was adopted to predict the top ten hub genes from the PPI network. As a result, AURKA, BUB1, CCNF, FEN1, FGF2, ITPKB, CDKN1A, TRIP13, KNTC1, and KIT were obtained (Fig. 4B). Among them, AURKA ranked the first hub gene in the PPI network, suggesting that AURKA played a highly important regulatory role in GC. As revealed by Fig. 5, AURKA, BUB1, CCNF, FEN1, TRIP13, and KNTC1 were markedly upregulated, whereas FGF2, ITPKB, CDKN1A, and KIT were remarkably reduced in GC tissues. Finally, a circRNA-miRNA-mRNA subnetwork (Fig. 6) was constructed based on the predicted top 10 hub genes.

### DEmRNAs mainly played a role in the signal transduction of GC and participated in the regulation of cell cycle

**Fig. 7:**
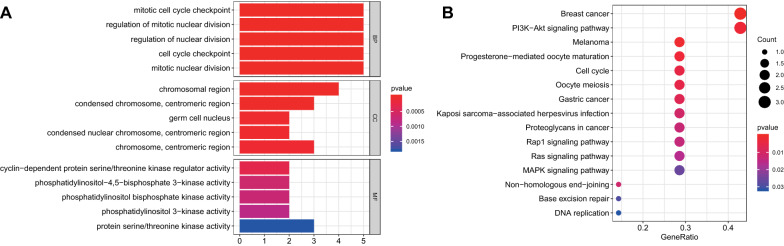
GO and KEGG pathway analyses of 8 DEmRNAs. **A** The histogram for the top 5 enriched GO terms in the biological process, cellular component, and molecular function. The X-axis indicates the number of enriched genes, and the Y-axis indicates the three categories of enriched GO terms. The redder the color is, the higher the enrichment degree is. **B** The bubble plot for the top 15 enriched KEGG pathways. The larger the circle is, the more the number of KEGG is; the redder the color is, the higher the enrichment degree is

In order to evaluate the functions of DEmRNAs in the ceRNA network on the occurrence and development of GC, we performed GO and KEGG functional enrichment analyses on the identified 10 DEmRNAs using the clusterProfiler package, with *p* < 0.05 as the threshold standard. GO enrichment analysis showed that the mRNAs related to biological processes were mainly enriched in biological processes including cell cycle checkpoints and mitotic mitosis. In terms of cell components, the mRNAs were mainly enriched in chromosomes, centromeres, germ cell nucleus and other cellular components. As for molecular function, the mRNAs were enriched in molecular functions such as protein serine/threonine kinase activity and phosphatidylinositol diphosphate kinase activity (Fig. 7A). KEGG analysis showed that the DEmRNA-related pathways mainly included various cancer pathways such as GC, PI3K-Akt signaling pathway, progesterone-mediated maturation of oocytes, cell cycle, etc. (Fig. 7B).

### circ_0061265 upregulated AURKA expression by competitively binding to miR-885-3p in GC

**Fig. 8:**
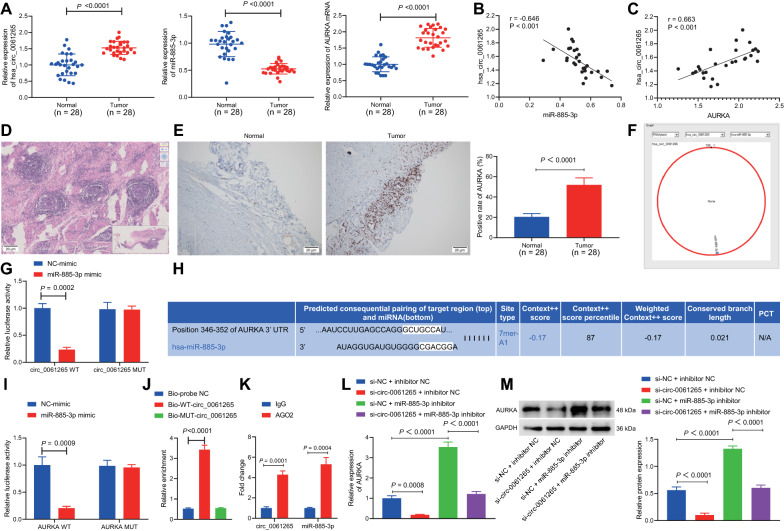
circ_0061265 upregulates AURKA expression by competitively binding to miR-885-3p in GC. **A** The expression of circ_0061265, miR-885-3p and AURKA in GC tissues and adjacent normal gastric tissues determined by RT-qPCR. n = 28. **B** Pearson correlation analysis of circ_0061265 and miR-885-3p expression in GC tissues. **C** Pearson correlation analysis of circ_0061265 and AURKA expression in GC tissues. **D** HE staining was used to observe the pathological changes of GC (scale bar: 20 μm). **E** Immunohistochemistry detection of AURKA expression in GC tissues and adjacent normal gastric tissues (scale bar: 20 μm). n = 28. **F** The binding site of circ_0061265 and miR-885-3p. **G** Dual luciferase gene reporter assay for verification of the binding relationship between circ_0061265 and miR-885-3p. **H** Binding site between miR-885-3p and AURKA 3'-UTR. **I** Dual luciferase gene reporter assay for verification of miR-885-3p and AURKA. **J** RNA-pull down to detect the enrichment of miR-885-3p, *compared with Bio-probe NC group, *p* < 0.05. **K** RIP detected the binding of circ_0061265 and miR-885-3p to AGO2, *p < 0.05 compared with IgG. **L** RT-qPCR detection of AURKA in GC cells treated with si-NC + inhibitor NC, si-circ-0061265 + inhibitor NC, si-NC + miR-885-3p inhibitor or si-circ-0061265 + miR-885-3p inhibitor. **M** Western blot analysis of protein expression of AURKA. *p < 0.05 between two groups

The promoting function of AURKA on tumor growth and cell survival has been signified [20]. Data from RT-qPCR displayed significantly higher expression of circ_0061265 and AURKA and lower miR-885-3p expression in GC tissues in relation to in the adjacent normal tissues (Fig. 8A). Pearson correlation analysis demonstrated a negative correlation between circ_0061265 and miR-885-3p, but a positive correlation between circ_0061265 and AURKA (Fig. 8B, C). Observation from HE staining revealed that nodal cells in GC tissue were arranged disorderly and infiltrated (Fig. 8D). Besides, the markedly elevated expression of AURKA in GC tissues was observed (Fig. 8E). Through bioinformatics database, the binding site between circ_0061265 and miR-885-3p was predicted (Fig. 8F) which was also confirmed through luciferase assay that the luciferase activity of GC cells expressing circ_0061265 WT after co-transfection with miR-885-3p mimic was significantly lower than that of the control, while no significant difference was seen after co-transfection of GC cells expressing circ_0061265 MUT with miR-885-3p mimic (Fig. 8G).

Binding sites between miR-885-3p and AURKA were also predicted through TargetScan database (Fig. 8H), which was validated by luciferase assay that after co-transfection with miR-885-3p-mimic, the luciferase activity in GC cells expressing AURKA 3’-UTR WT showed a significant decline compared to that in the control; however, co-transfection of AURKA 3′-UTR MUT and miR-885-3p-mimic didn’t change the luciferase activity of GC cells (Fig. 8I).

RNA-pull down experiment showed that compared with the Bio-probe NC group, there was no significant difference in the miR-885-3p enrichment in the Bio-MUT-circ_0061265 group, and the enrichment of miR-885-3p in the Bio-WT-circ_0061265 group was significantly increased, which indicated that Bio-Wt-circ_0061265 could promote the enrichment of miR-885-3p (Fig. 8J). RIP experiment showed that compared with IgG, the binding of AGO2 to circ_0061265 was significantly increased, suggesting that circ_0061265 could bind to AGO2 protein, that is, miR-885-3p and circ_0061265 could directly bind (Fig. 8K).

Furthermore, we detected whether circ_0061265 regulates AURKA by regulating miR-885-3p. As reflected by Fig. 8L, M downregulated circ_0061265 reduced the AURKA mRNA and protein expression. Downregulation of miR-885-3p contributed to notably increased mRNA and protein expression of AURKA. miR-885-3p inhibitor partially rescued the effects of si-circ_0061265 on AURKA. Coherently, circ_0061265 was capable of increasing AURKA expression by competitively binding to miR-885-3p.

### Silencing AURKA suppressed GC cell malignant features and chemoresistance

**Fig. 9:**
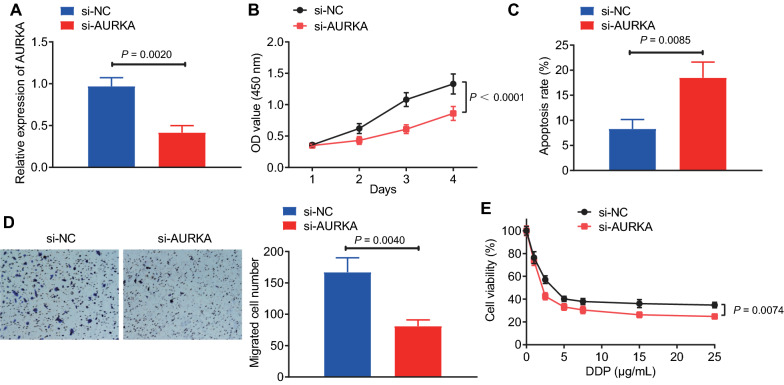
Silencing AUKRA suppresses GC cell proliferation, migration, and chemoresistance. **A** RT-qPCR detection of transfection efficiency of silenced AURKA in NCI-N87 cells. **B** CCK-8 assay was applied to evaluate cell proliferation. **C** Flow cytometry was used to detect cell apoptosis. **D** Transwell was used to detect cell migration (scale bar: 50 μm). **E** Detection of cell viability under different concentrations of cisplatin. *p < 0.05 between two groups. All experiments were repeated for three times

In order to further study the effect of AURKA on GC cells, we silenced AURKA in NCI-N87 cells (Fig. 9A). As reflected by Fig. 9B–D, silencing AURKA could inhibit the proliferation and migration of NCI-N87 cells, and promote cell apoptosis. In addition, silencing AURKA also inhibited cell viability after cisplatin treatment (Fig. 9E). Conclusively, silencing AURKA inhibited the proliferation, migration, and chemotherapy resistance of GC cells and promoted their apoptosis.

### circ_0061265 promoted malignant transformation and chemoresistance of GC by regulating AURKA through miR-885-3p

**Fig. 10:**
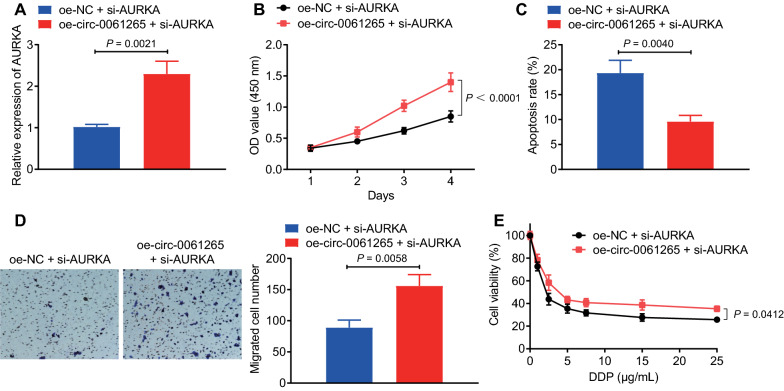
Effects of circ_0061265 on the proliferation, apoptosis, migration and chemotherapy resistance of GC cells by regulating AURKA via miR-885-3p. **A** RT-qPCR detection of AURKA expression in NCI-N87 cells. **B** CCK-8 assay was applied to evaluate cell proliferation. **C** Flow cytometry was used to detect cell apoptosis. **D** Transwell was used to detect cell migration (scale bar: 50 μm). **E** Detection of cell viability under different concentrations of cisplatin. *p < 0.05 between two groups. All experiments were repeated for three times

To verify that circ_0061265 affects GC cell function by regulating AURKA via miR-885-3p, we simultaneously intervened circ_0061265 and AURKA in NCI-N87 cells. It was demonstrated that relative to oe-NC + si-AURKA treatment, oe-circ_0061265 + si-AURKA treatment led to enhanced AURKA (Fig. 10A). Additionally, oe-circ_0061265 + si-AURKA treatment caused increased proliferation and migration but reduced apoptosis relative to oe-NC + si-AURKA treatment (Fig. 10B–D). Further, the viability of cells in the oe-circ_0061265 + si-AURKA group was significantly increased after cisplatin treatment (Fig. 10E). Therefore, circ_0061265 could reverse the inhibitory effect of silencing AURKA on the proliferation, migration and chemotherapy resistance of GC cells.

## Discussion

As microarray and RNA sequencing technologies develop, an increasing number of circRNAs have been discovered and suggested to be of vital functionality in a wide range of human diseases [21], including GC [22]. It remains surprisingly limited about their ceRNA regulatory network in GC. Herein, we performed integrative analysis of GEO and TCGA data to unveil circRNA-mediated ceRNA regulatory network in GC and performed in vitro experiments to validate the ceRNA network.

Based on GEO and TCGA databases, we initially screened 13 DEcircRNAs, 241 DEmiRNAs and 7483 DEmRNAs related to GC. Furthermore, we discovered that 9 circRNAs (circ_0061265, hsa_circ_0008035, hsa_circ_0061274, hsa_circ_0023642, hsa_circ_0000026, hsa_circ_0077248, hsa_circ_0068610, hsa_circ_0040039 and hsa_circ_0005927) participated in the ceRNA network of GC, among which circ_0061265 was of highly importance. Some studies have reported abnormal expression patterns of circRNAs is in relation to the pathogenesis and prognosis of GC, suggesting that circRNA can be used as a tumor-related biomarker. For instance, hsa_circ_0008035 was found amplified in GC, and siRNA knockdown of it was sufficient to retard GC cell proliferation and invasion [23]. Huang et al*.* [24] revealed downregulated hsa_circ_0000026 in 3 GC tissue samples in relation to normal gastric tissue samples. Additionally, Zhang et al. [25] unfolded that circDLST, which was upregulated in GC tissues alongside the cell lines, played a contributory role to GC tumorigenesis and metastasis and was related to patient survival. Besides, Rong et al. reported that circPSMC3 could combine with miR-296-5p, then positively regulating the expression of PTEN, and suppressing the proliferative and metastatic potentials of GC [26]. However, no previous functional evidence of circ_0061265 in human cancers has been documented.

In addition, ten hub genes were respectively obtained by topological analysis using MCC, including AURKA, BUB1, CCNF, FEN1, FGF2, ITPKB, CDKN1A, TRIP13, KNTC1 and KIT. Of note, AUKRA was ranked the first among these genes in the PPI network and showed significant high expression according to the results from RT-qPCR and immunohistochemistry. To our acknowledge, the involvement of AUKRA in the development of GC has been unfolded. For example, downregulation of AURKA by gossypin could lead to suppression of GC growth [27]. Besides, significant overexpression of AURKA was revealed in human GC samples, the inhibition of which using an investigational small-molecule specific inhibitor, alisertib, could markedly decrease the in vitro cell survival and in vivo xenograft tumor growth by diminishing the HDM2 protein level and inducing P53 transcriptional activity [20]. Moreover, Hou et al*.* found that inhibition of AURKA could result in more intensive apoptosis in GC by repressing p27 inhibition on Bax cleavage [28]. These reports are supportive of our finding regarding the oncogenic role of AURKA in GC progression.

In the subsequent analysis, we elucidated the impact of circ_0061265-miRNA-885-3p-AURKA ceRNA network in GC, which demonstrated that circ_0061265 promoted the occurrence and development of GC by competitively binding to miRNA-885-3p to regulate the expression of AUKRA. A circRNA can bind with a miRNA, commonly known as “miRNA sponge”, which can reduce the cytoplasmic level of miRNA and release their respective downstream target mRNA, thus exhibiting tumor-suppressive [29] or tumor-promoting [30] effects on human cancer. In the present study, RT-qPCR and dual luciferase gene reporter assay displayed that miRNA-885-3p could targetedly regulate AURKA in GC. Consistently, miRNA-885-3p was found to decrease the expression of AURKA, which suppressed docetaxel chemoresistance in lung adenocarcinoma [16]. It is noteworthy that miRNA-885-3p could decrease the expression of cyclin-dependent kinase 4 at the post-transcriptional level, thereby inhibiting GC cell proliferation [15]. miRNA-885-3p could also disrupt angiogenesis through regulation of BMPR1A and repression of BMP/Smad/Id1 signaling, thereby suppressing the growth of colon cancer cell xenografts [31]. Intriguingly, another study unveiled that miRNA-885-5p could modulate YPEL1 to accelerate the proliferation and invasion in GC [32]. Importantly, there are many miRNAs involved in the regulation of AURKA expression [33–36]. In our study, it is demonstrated that the circ_0061265-miRNA-885-3p-AURKA ceRNA network could affect the progression of GC.

Due to the limitations of experimental time and research funding, we have not been able to conduct in-depth in vitro cell experiments to explore the effect of the circ_0061265/miR-885-3p/AURKA co-expression regulatory network on the proliferation, migration and invasion of gastric cancer cells in vitro, which may limit the scientific value of our study. However, we will try to perfect the deficiencies of this research in future research.

## Conclusion

**Fig. 11:**
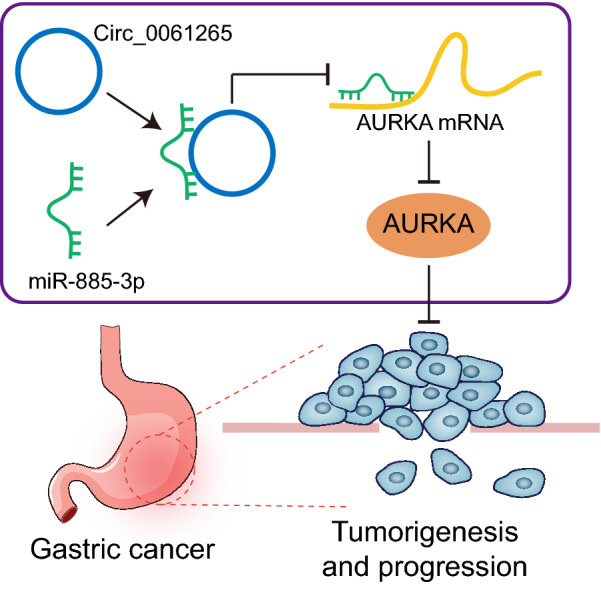
The molecular mechanism plot for the role of circ 0061265-miR-885-3p-AUKRA ceRNA network in the occurrence and development of GC. miR-885-3p is significantly downregulated while circ_0061265 and AUKRA were upregulated in GC. circ_0061265 can promote the occurrence and development of GC by competitively binding to miR-885-3p to regulate the expression of AUKRA. The circ_0061265-miR-885-3p-AUKRA ceRNA network may be a key pathway involved in the occurrence and development of GC

In conclusion, circ_0061265 can promote the occurrence and development of GC, which is achieved by competitively binding to miR-885-3p to modulate AUKRA expression (Fig. 11). Our work provides an enhanced understanding of the circRNA-mediated ceRNA network in GC. Nevertheless, further validation of the network in GC initiation or progression is needed.

## Supplementary Information

**Additional file 1**: **Table S1** RT-qPCR primer sequences (human). **Table S2** Basic characteristics of the 13 differently expressed circRNAs.

**Additional file 2: Fig. S1.** The structure of the 9 DEcircRNAs in the GSE78092 dataset. (**A**) hsa_circ_0061265. (**B**) hsa_circ_0008035. (**C**) hsa_circ_0061274. (**D**) hsa_circ_0023642. (**E**) hsa_circ_0000026. (**F**) hsa_circ_0077248. (**G**) hsa_circ_0068610. (**H**) hsa_circ_0040039. (**I**) hsa_circ_0005927.

## Data Availability

The authors confirm that the data supporting the findings of this study are available within the article.
