# Supplementary material for: circ_0061265 competitively binds to microRNA-885-3p to promote the development of gastric cancer by upregulating AURKA expression
Source: Cancer Cell Int. 2022 Sep 5;22:277. doi: 10.1186/s12935-022-02646-3 (PMC9446739; doi:10.1186/s12935-022-02646-3)
Supplement: Supplementary file 1 — Additional file 1: Table S1 RT-qPCR primer sequences (human). Table S2 Basic characteristics of the 13 differently expressed circRNAs. [file 12935_2022_2646_MOESM1_ESM.docx]

**Table S1** RT-qPCR primer sequences (human)

| Genes | Primer sequences (5’-3’) |
| --- | --- |
| miR-885-3p | Forward: AGGCAGCGGGGTGTAGTGGATA |
|  | Reverse: Universal reverse primer |
| U6 | Forward: CTCGCTTCGGCAGCACA |
|  | Reverse: AACGCTTCACGAATTTGCGT |
| circ_0061265 | Forward: CTGCCATCTAACTCCGCTGA |
|  | Reverse: GTCTGTCCGCTTCCGTCC |
| AURKA | Forward: AGTGACCACTCTGCCCTGAC |
|  | Reverse: CGGACAGACACACAGCATTCC |
| GAPDH | Forward: GCACCGTCAAGGCTGAGAAC |
|  | Reverse: ATGGTGGTGAAGACGCCAGT |

**Table S2** Basic characteristics of the 13 differently expressed circRNAs

| circRNA ID | Position | Genomic length | Strand | Best transcript | Gene symbol | Regulation |
| --- | --- | --- | --- | --- | --- | --- |
| circ_0061265 | [chr21:15456270-15456465](http://genome.mdc-berlin.de/cgi-bin/hgTracks?db=hg19&position=chr21:15456270-15456465&hubUrl=http://bimsbstatic.mdc-berlin.de/hubs/rajewsky/circBase/hub.txt) | 195 | + | None | None | Up |
| hsa_circ_0008035 | [chr8:118830673-118849440](http://genome.mdc-berlin.de/cgi-bin/hgTracks?db=hg19&position=chr8:118830673-118849440&hubUrl=http://bimsbstatic.mdc-berlin.de/hubs/rajewsky/circBase/hub.txt) | 18767 | - | [NM_000127](http://www.ncbi.nlm.nih.gov/nuccore/NM_000127) | [EXT1](http://www.ncbi.nlm.nih.gov/gene/?term=2131) | Up |
| hsa_circ_0061274 | [chr21:16386664-16386787](http://genome.mdc-berlin.de/cgi-bin/hgTracks?db=hg19&position=chr21:16386664-16386787&hubUrl=http://bimsbstatic.mdc-berlin.de/hubs/rajewsky/circBase/hub.txt) | 123 | - | [NM_003489](http://www.ncbi.nlm.nih.gov/nuccore/NM_003489) | [NRIP1](http://www.ncbi.nlm.nih.gov/gene/?term=8204) | Up |
| hsa_circ_0000144 | [chr1:160472466-160472794](http://genome.mdc-berlin.de/cgi-bin/hgTracks?db=hg19&position=chr1:160472466-160472794&hubUrl=http://bimsbstatic.mdc-berlin.de/hubs/rajewsky/circBase/hub.txt) | 328 | + | [NM_052931](http://www.ncbi.nlm.nih.gov/nuccore/NM_052931) | [SLAMF6](http://www.ncbi.nlm.nih.gov/gene/?term=114836) | Up |
| hsa_circ_0023642 | [chr11:75727858-75728024](http://genome.mdc-berlin.de/cgi-bin/hgTracks?db=hg19&position=chr11:75727858-75728024&hubUrl=http://bimsbstatic.mdc-berlin.de/hubs/rajewsky/circBase/hub.txt) | 166 | + | [NM_003369](http://www.ncbi.nlm.nih.gov/nuccore/NM_003369) | [UVRAG](http://www.ncbi.nlm.nih.gov/gene/?term=7405) | Up |
| hsa_circ_0000026 | [chr1:21377358-21437876](http://genome.mdc-berlin.de/cgi-bin/hgTracks?db=hg19&position=chr1:21377358-21437876&hubUrl=http://bimsbstatic.mdc-berlin.de/hubs/rajewsky/circBase/hub.txt) | 60518 | - | [NM_001198803](http://www.ncbi.nlm.nih.gov/nuccore/NM_001198803) | [EIF4G3](http://www.ncbi.nlm.nih.gov/gene/?term=8672) | Down |
| hsa_circ_0077248 | [chr6:86281854-86282092](http://genome.mdc-berlin.de/cgi-bin/hgTracks?db=hg19&position=chr6:86281854-86282092&hubUrl=http://bimsbstatic.mdc-berlin.de/hubs/rajewsky/circBase/hub.txt) | 238 | - | [NM_153816](http://www.ncbi.nlm.nih.gov/nuccore/NM_153816) | [SNX14](http://www.ncbi.nlm.nih.gov/gene/?term=57231) | Down |
| hsa_circ_0001561 | [chr5:177652366-177652583](http://genome.mdc-berlin.de/cgi-bin/hgTracks?db=hg19&position=chr5:177652366-177652583&hubUrl=http://bimsbstatic.mdc-berlin.de/hubs/rajewsky/circBase/hub.txt) | 217 | - | [NM_153373](http://www.ncbi.nlm.nih.gov/nuccore/NM_153373) | [AGXT2L2](http://www.ncbi.nlm.nih.gov/gene/?term=85007) | Down |
| hsa_circ_0092341 | [chr6:42071996-42072196](http://genome.mdc-berlin.de/cgi-bin/hgTracks?db=hg19&position=chr6:42071996-42072196&hubUrl=http://bimsbstatic.mdc-berlin.de/hubs/rajewsky/circBase/hub.txt) | 200 | - | [NM_001164446](http://www.ncbi.nlm.nih.gov/nuccore/NM_001164446) | [C6orf132](http://www.ncbi.nlm.nih.gov/gene/?term=647024) | Down |
| hsa_circ_0068610 | [chr3:195785154-195787118](http://genome.mdc-berlin.de/cgi-bin/hgTracks?db=hg19&position=chr3:195785154-195787118&hubUrl=http://bimsbstatic.mdc-berlin.de/hubs/rajewsky/circBase/hub.txt) | 1964 | - | [NM_003234](http://www.ncbi.nlm.nih.gov/nuccore/NM_003234) | [TFRC](http://www.ncbi.nlm.nih.gov/gene/?term=7037) | Down |
| hsa_circ_0040039 | [chr16:69279504-69318147](http://genome.mdc-berlin.de/cgi-bin/hgTracks?db=hg19&position=chr16:69279504-69318147&hubUrl=http://bimsbstatic.mdc-berlin.de/hubs/rajewsky/circBase/hub.txt) | 38643 | + | [NM_006750](http://www.ncbi.nlm.nih.gov/nuccore/NM_006750) | [SNTB2](http://www.ncbi.nlm.nih.gov/gene/?term=6645) | Down |
| hsa_circ_0005927 | [chr8:42259305-42260979](http://genome.mdc-berlin.de/cgi-bin/hgTracks?db=hg19&position=chr8:42259305-42260979&hubUrl=http://bimsbstatic.mdc-berlin.de/hubs/rajewsky/circBase/hub.txt) | 1674 | + | [NM_001135694](http://www.ncbi.nlm.nih.gov/nuccore/NM_001135694) | [VDAC3](http://www.ncbi.nlm.nih.gov/gene/?term=7419) | Down |
| hsa_circ_0041732 | [chr17:6350782-6351078](http://genome.mdc-berlin.de/cgi-bin/hgTracks?db=hg19&position=chr17:6350782-6351078&hubUrl=http://bimsbstatic.mdc-berlin.de/hubs/rajewsky/circBase/hub.txt) | 296 | + | [NM_019013](http://www.ncbi.nlm.nih.gov/nuccore/NM_019013) | [FAM64A](http://www.ncbi.nlm.nih.gov/gene/?term=54478) | Down |
